# Noise insertion in CT for cocaine body packing: where is the limit of extensive dose reduction?

**DOI:** 10.1186/s40001-018-0356-3

**Published:** 2018-12-07

**Authors:** Joel Aissa, Edwin Bölke, Lino M. Sawicki, Elisabeth Appel, Christoph Thomas, Philipp Heusch, Martin Sedlmair, Karl Krzymyk, Patric Kröpil, Gerald Antoch, Johannes Boos

**Affiliations:** 1 Department of Diagnostic and Interventional Radiology, Medical Faculty, University Dusseldorf, Moorenstr. 5, 40225 Dusseldorf, Germany; 2 Department of Radiation Oncology, Medical Faculty, University Dusseldorf, Moorenstr. 5, 40225 Dusseldorf, Germany; 3 Computed Tomography, Siemens Healthineers GmbH, Forchheim, Germany; 4 Department of Radiology, BG Klinikum Duisburg gGmbH, 47249 Duisburg, Germany

**Keywords:** CT dose, Image analysis, Drug abuse, Low dose CT

## Abstract

**Background:**

To evaluate the detection rate and image quality in CT-body-packer-screening at different radiation-dose levels and to determine a dose threshold that enables a reliable detection of incorporated body packs and incidental findings with a maximum of dose saving.

**Materials and methods:**

We retrospectively included 27 individuals who underwent an abdominal CT with automated exposure control due to suspected body packing. CT images were reconstructed at different radiation-dose levels of 50%, 10, 5% and 1% using iterative reconstructions. All 135 CT reconstructions were evaluated by three independent readers. Reviewers determined the presence of foreign bodies and evaluated the image quality using a 5-point ranking scale. In addition, visualization of incidental findings was assessed.

**Results:**

A threshold of 5% (effective dose 0.11 ± 0.07 mSv) was necessary to correctly identify all 27 patients with suspected body packing. Extensive noise insertion to a dose level of 1% (0.02 ± 0.01 mSV) led to false-positive solid cocaine findings in three patients. Image quality was comparable between 100 and 50%. The threshold for correct identification of incidental findings was 10% of the initial dose (effective dose 0.21 ± 0.13 mSv).

**Conclusions:**

Our results indicate that dose of abdominal CT for the detection of intracorporeal cocaine body packets can be markedly reduced to up to 5% of the initial dose while still providing sufficient image quality to detect ingested body packets. However, a minimum effective dose of 0.21 mSv (10% of initial dose) seems to be required to properly identify incidental findings.

## Introduction

Concealment and transportation of cocaine is a growing business with worldwide impact and the transportation of drug containers by ingestion (“body packing”) is a commonly used form of worldwide drug smuggling [1, 2]. Cocaine is not the only drug concealed by body packers, however; due to its price, cocaine is still the most frequently transported drug [3]. Leaky drug containers may lead to cocaine overdose, thus a fast and accurate detection of body packets is required [4]. Abdominal plain radiography and CT localizer images are of limited value in the detection of body packets due to a low sensitivity [5, 6]. Because of its high sensitivity, computed tomography (CT) is the first line imaging modality in the detection of those drug couriers [5, 6]. However, radiation exposure from a regular abdominal CT is considerably higher than from plain radiography and thus is a critical factor as body packers are usually young individuals [3, 7]. Therefore, dose optimization is important in this special setting, and initial studies on dose optimization in body packing CT reported a dose reduction while retaining diagnostic image quality (IQ) [5, 8]. However, the limit of extensive dose reduction in the setting of body packing was only investigated in experimental animal studies and cadavers but not in clinical studies with human individuals [9, 10].

The aim of our study was to evaluate the image quality, the diagnostic yield for body packets and the accuracy for the detection of incidental findings in suspected body packer abdominal CT to determine a dose threshold that enables reliable detection of incorporated body packets and incidental findings with a maximum of dose saving.

## Patient, materials and methods

### Patient population

This retrospective study was approved by the local ethics committee (IRB number: 5652). Twenty-seven individuals (20 male, 7 female) with a mean age of 37.8 ± 11.1 years (range 19–58 years) underwent a CT for suspected body packing between November 2014 and February 2016. All CTs were requested by the local customs authorities.

### Computed tomography protocol

All CT examinations were performed on a 128-row dual source CT scanner using the single-source mode (Somatom Definition Flash, Siemens Healthineers, Erlangen, Germany). Scans were performed with a fixed tube voltage of 80 kVp and automated tube current modulation (CarekV semi-mode, Siemens Healthineers) with a reference tube current time product of 60 mAs. The scan volume included the basal lungs to the proximal femur. Rotation time was 0.5 s and collimation was 0.6 mm. Image quality of the CT protocol has been previously validated [11]. All scans were performed without oral or intra venous contrast media.

### Image reconstructions and processing

Reconstructions were performed using an offline workstation and a prototype software device (ReconCT 13.8.2.0, Siemens Healthineers). Raw data is required to perform noise-insertion reconstructions. The system was calibrated prior to the reconstruction process by scanning a 20 cm water phantom with the same scan mode that is used for suspected body packer. The calibration data were transferred into the ReconCT software and defined as the standard for the noise simulation process. Reconstructions with simulated dose levels of 100%, 50%, 10%, 5% and 1% of initial dose were performed using a validated noise-insertion tool (RawData Noise Insertion, ReconCT 13.8.2.0, Siemens Healthineers) [12]. All reconstructions were performed with iterative reconstruction (SAFIRE, Level 3) in axial orientation (3-mm slice thickness) using a medium smooth kernel (I30f) and a sharp kernel (I70f).

### Subjective image quality and pack identification

All reconstructions were loaded onto an Advantage Windows Workstation (Fujitsu, Tokyo, Japan) and axial images were reviewed using a Digital Imaging and Communications in Medicine (DICOM) viewer software (VISI, 1.10.03, Siemens Healthineers). Evaluation was performed by three independent readers (xxx, yyy, and zzz) with 1, 6, and 8 years of experience in radiology, and all three readers were competent in the detection of solid and liquid body packs. The readers determined the presence and composition of foreign bodies (liquid or solid). The initial 100% dose CT examination and the clinical CT reports served as reference standard. In cases positive on CT, we received a stool analysis, which allowed confirmation of solid or liquid content. Readers evaluated the reduced dose reconstructions across all patients in a random order. Readers were blinded to the presence of body packets on the 100% reconstructions during the review. The 100% reconstructions were evaluated after review of the reduced dose reconstructions in a random order.

The confidence level for positive or negative body packing was evaluated using a 3-point ranking scale (1 = low confidence in the diagnosis; 2 = moderate confidence in the diagnosis; 3 = excellent confidence in the diagnosis). The image quality was evaluated based on visualization of important structures as defined by the European Quality Criteria [8, 13] using a 5-point ranking scale (1 = excellent image quality; 2 = good image quality; 3 = moderate image quality; 4 = poor image quality; 5 = nondiagnostic). Image quality was scored separately for the proper visualization of the liver parenchyma, the splenic parenchyma, the intestine, the perivascular retroperitoneal space, the pancreatic contours, the duodenum, the kidneys, the aorta, and the vena cava [13]. Mean attenuation of body packets (HU) were analyzed by 3 ROI measurements in all positive cases.

### Identification of secondary findings

Secondary findings were defined as all findings that were not related to the body packing. Original reports were screened for secondary findings by aaa and reviewed in the 100% reconstructions (radiologist with 6 years of experience). There were twelve incidental findings in nine patients (Aortic vasosclerosis *n* = 3; nephrolithiasis *n* = 1; accessory spleen *n* = 2; spondylolysis *n* = 1; subcutaneous atheroma *n* = 1; dysplasia of the hips *n* = 1, liver cyst *n* = 1, partial gastrectomy *n* = 1; butterfly swirls *n* = 1). Two radiologists (yyy and zzz) with 6 and 8 years of experience in radiology, who were blinded to the clinical data reviewed the 108 CT reconstructions (dose levels 50%, 10%, 5%, and 1%) of the 27 patients for secondary findings in a random order. The 100% reconstructions were reviewed in a random order afterward.

Axial reconstructions with a smooth kernel (I30f) and a sharp kernel (I70f) were provided.

## Calculation of radiation dose

Volumetric computed tomography dose index (CTDI_vol_) and Dose Length Product (DLP) were extracted from the Picture Archiving and Communication System (PACS). To estimate the effective radiation dose, DLP values were converted to Millisieverts (mSv) by using conversion factors provided by the American Association of Physicists in Medicine (AAPM) for abdominal CT examinations (0.015 mSv/mGycm) [14]. To estimate the potential dose saving, the effective dose at the simulated dose levels (50%, 10%, 5%, and 1% of initial effective dose) was calculated. Patient`s constitution was evaluated by abdominal diameter measurements. Therefore, we analyzed the lateral (Dlat) and anterior to posterior diameter (Dap) at the level of largest diameter in abdominal axial slices. Measurements were performed by an independent reader (zzz) with 8 years of experience in radiology. The effective Diameter (Deff) was calculated using the obtained Dlat and Dap [15].

$$ {\text{Deff}}\, = \,\sqrt {\left( {{\text{Dlat}}\, \times \,{\text{Dap}}} \right)} $$

## Statistical analysis

IBM SPSS Statistics 21 for Windows (IBM, SPSS Statistics 21, Chicago, IL) was used for statistical analysis. Values for subjective image quality are reported as media*n* ± interquartile range (IQR). A Kolmogorov-test was performed to test for normality. A Wilcoxon test was used as a nonparametric test for paired values. A Chi-square test was performed to compare the different groups. Kappa-value was calculated to evaluate the interobserver agreement. Interobserver agreement was defined as excellent (*κ* > 0.80), good (*κ* = 0.61–0.80), moderate (*κ* = 0.41–0.60), fair (*κ* = 0.21–0.40), and poor (k ≤ 0.20) [16]. Level of statistical significance was set to < 0.05.

## Results

### Radiation dose

CTDI_vol_ and DLP of our standard institutional CT protocol for the detection of body packets were 2.96 ± 1.83 mGy (range 1.09–8.94 mGy) and 140.0 ± 88.1 mGycm (range 49–393 mGycm). The effective dose was 2.10 ± 1.32 mSv (range 0.74–5.89 mSv). The mean tube current was 150.7 ± 93.5 mAs. The effective doses of the 50%, 10%, 5%, and 1% reconstructions are shown in Table 1. The mean Dlat was 34.2 ± 5.8 cm (range 48.4–27.6 cm) and the mean Dap was 25.7 ± 5.9 cm (range 35.7–17.3 cm). The mean Deff was 29.9 ± 5.9 cm (range 22.4–39.1).

**Table 1:** The overall estimated dose results (CTDI_vol_, DLP, and effective dose), due to dose level at 100% and the estimated dose savings at 50%, 10, 5%, and 1% of initial dose

	CTDI_vol_ (mGy) mean ± SD	DLP (mGycm) mean ± SD	Eff. dose (mSv) mean ± SD
Full dose (%)			
100	2.96 ± 1.83	140 ± 88.1	2.10 ± 1.32
Estimated dose savings (%)			
50			1.05 ± 0.66
10			0.21 ± 0.13
5			0.11 ± 0.07
1			0.02 ± 0.01

### Body pack identification

At the dose level of 100% the three observers detected incorporated body packets in 8/27 individuals (29.6%). All patients with body packets hat swallowed multiple body packets (> 30 packets per patient). All readers correctly identified liquid (1/8, 12.5%) and solid (7/8, 87.5%) body packets.

**Fig. 1:**
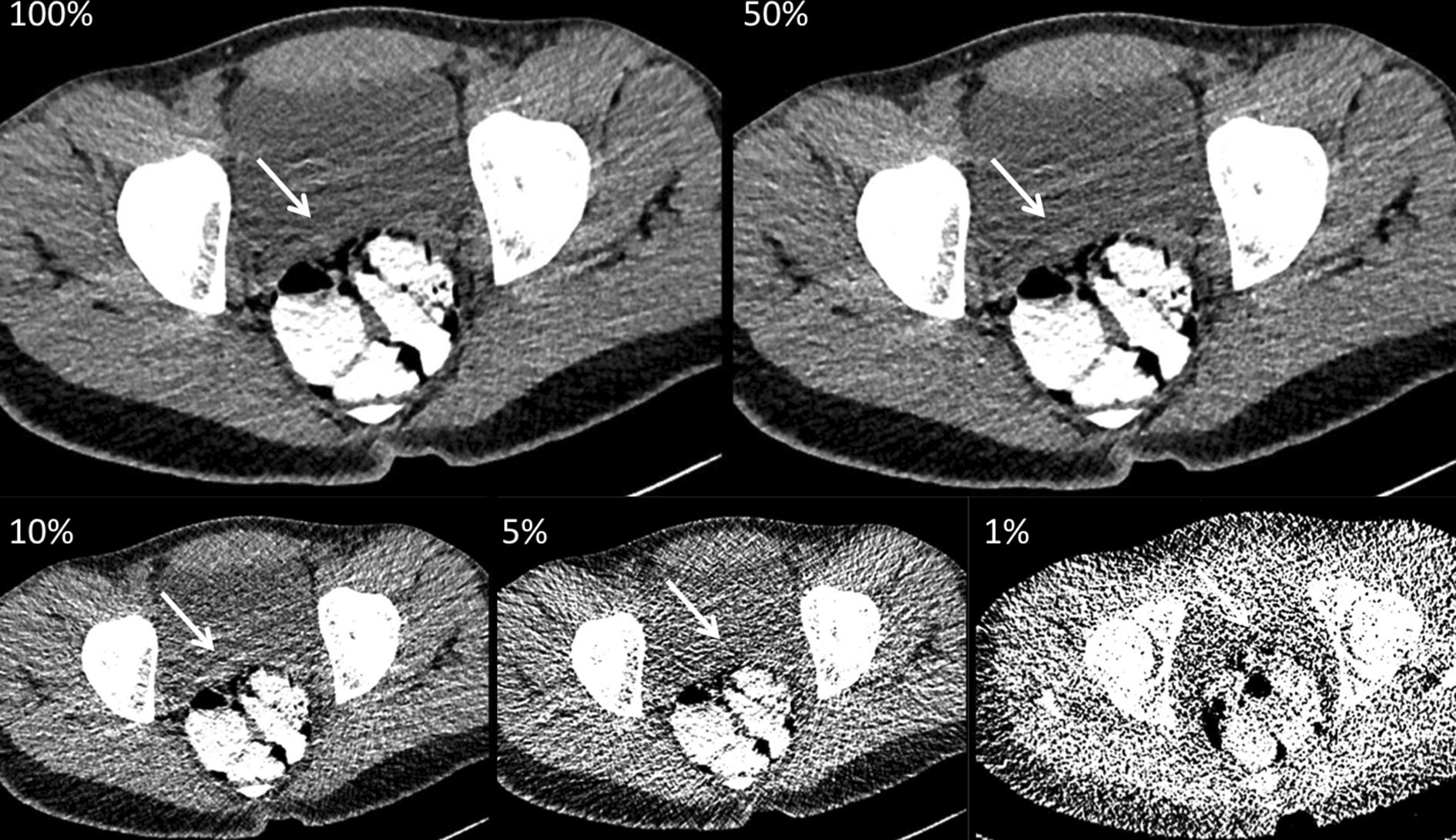
A 22-year-old body packer, who was examined with 80 kVp and automated tube current modulation (tube current time product: 88 mAs, effective dose at 100%: 1.16 mSv). The axial reconstructions (soft tissue window, window level 40/300) with stepwise reduced dose levels (100%, 50%, 10%, 5%, and 1%) show multiple liquid body packets (white arrows) which are hyperattenuating compared to the surrounding bowel content. While differentiation of bowel and that of urinary bladder decrease within stepwise dose reduction, liquid cocaine remains easily detectable with only 1% of initial radiation dose (effective dose: 0.01 mSv)

**Fig. 2:**
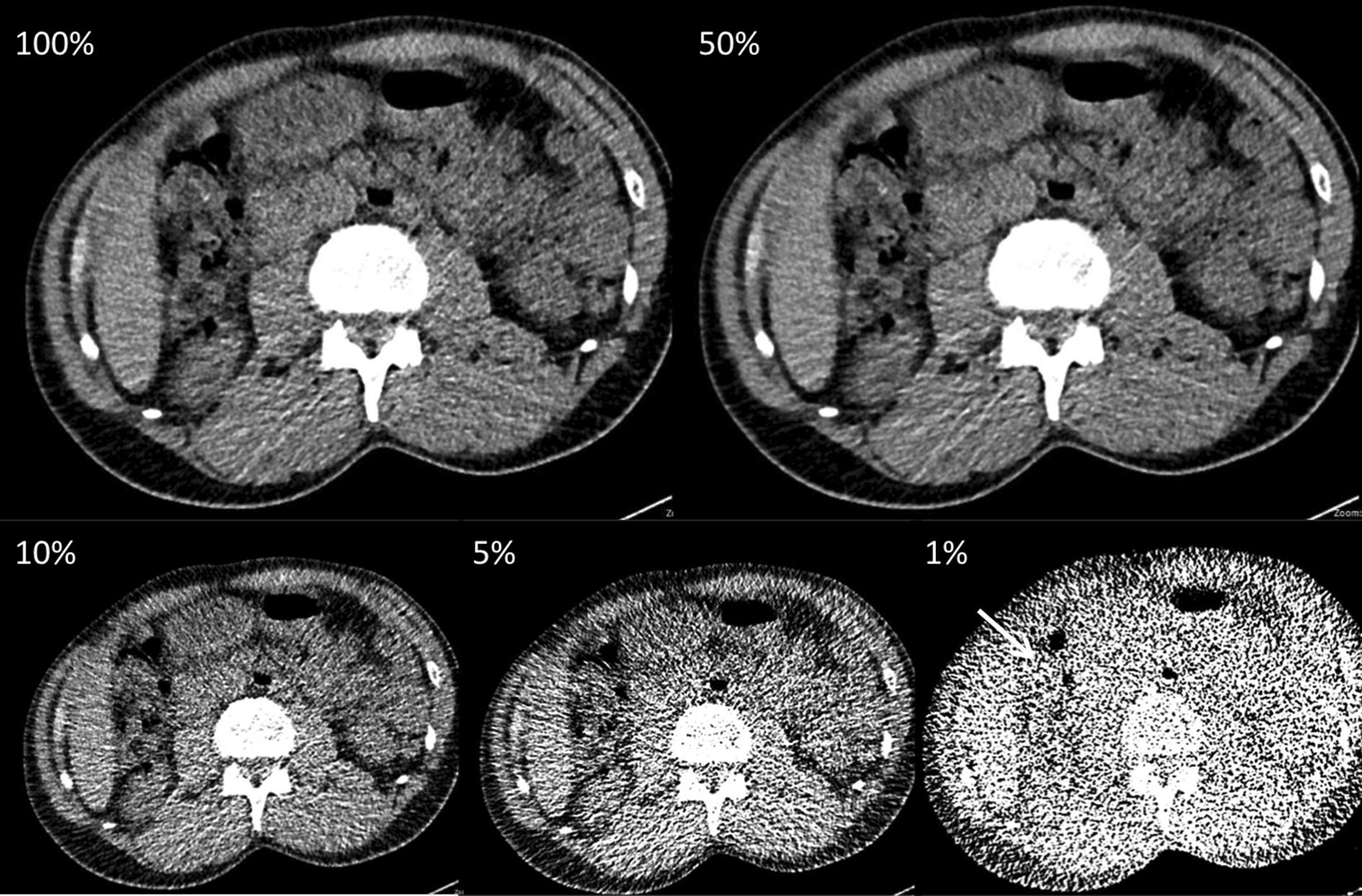
A 58-year-old individual suspected of body packing. CT examination was performed at 80 kVp with ATCM (tube current time product: 81 mAs, dose level 100% effective dose at 100%: 1.09 mSv). Axial CT scans (soft tissue window, window level 40/300) with stepwise dose reduction (100%, 50%, 10%, 5%, and 1%). In this case at 1% of initial dose (0.02 mSv), one additional false-positive solid body packet was rated by reader one (white arrow)

All body packets were properly identified at a dose level of 50%, 10%, and 5% (Fig. 1). However, one reader falsely classified one body packet as solid instead of liquid at a dose level of 5%. We found an excellent interobserver agreement of all three readers for 100%, 50%, and 10% (*κ* = 1). For 5%, we found a *κ* value of 0.91 between reader 1 and 2. At 1% of the initial dose, false-positive findings led to a significantly higher number of detected body packer cases (body packing cases: 8/27, 29.6%; reader 1: 8/27, 29.6%, reader 2: 9/27, 33.3%, reader 3: 10/27, 37.0%; *κ* reader 1 vs. 2 = 0.17, *κ* value reader 1 vs. 3 = 0.91, *κ* value reader 2 vs. 3 = 0.11; p = 0.03) (Fig. 2). The mean density of body packets was 292.2 ± 69.7 HU (range 391.3–157.7).

Concerning the confidence level for positive or negative body packing, we found no differences between 100% and all other dose levels (dose level 100%: median 3 ± 2; dose level 50%: median 3 ± 1, p = 0.73; dose level 10%: median 3 ± 1, p = 0.85; dose level 5%: median 3 ± 1, p = 0.47; dose level 1%: 3 ± 1.5, p = 0.34).

### Subjective image quality

Subjective image quality was significantly reduced for all simulated dose levels compared to the reference standard (dose level 100%: median 1 ± 2; dose level 50%: median 2 ± 2.5; p = 0.023; dose level 10%: median 3 ± 2, p < 0.0001; dose level 5%: median 4 ± 2, p < 0.0001; dose level 1%: median 5 ± 2, p < 0.0001). At 100% and 50% of radiation dose, all CT scans were rated diagnostic while, at 10%, 5%, and 1%, all three readers rated several CT scans as nondiagnostic (10%: *n* = 13; 5%: *n* = 24; 1%: *n* = 42).

### Identification of secondary findings

**Fig. 3:**
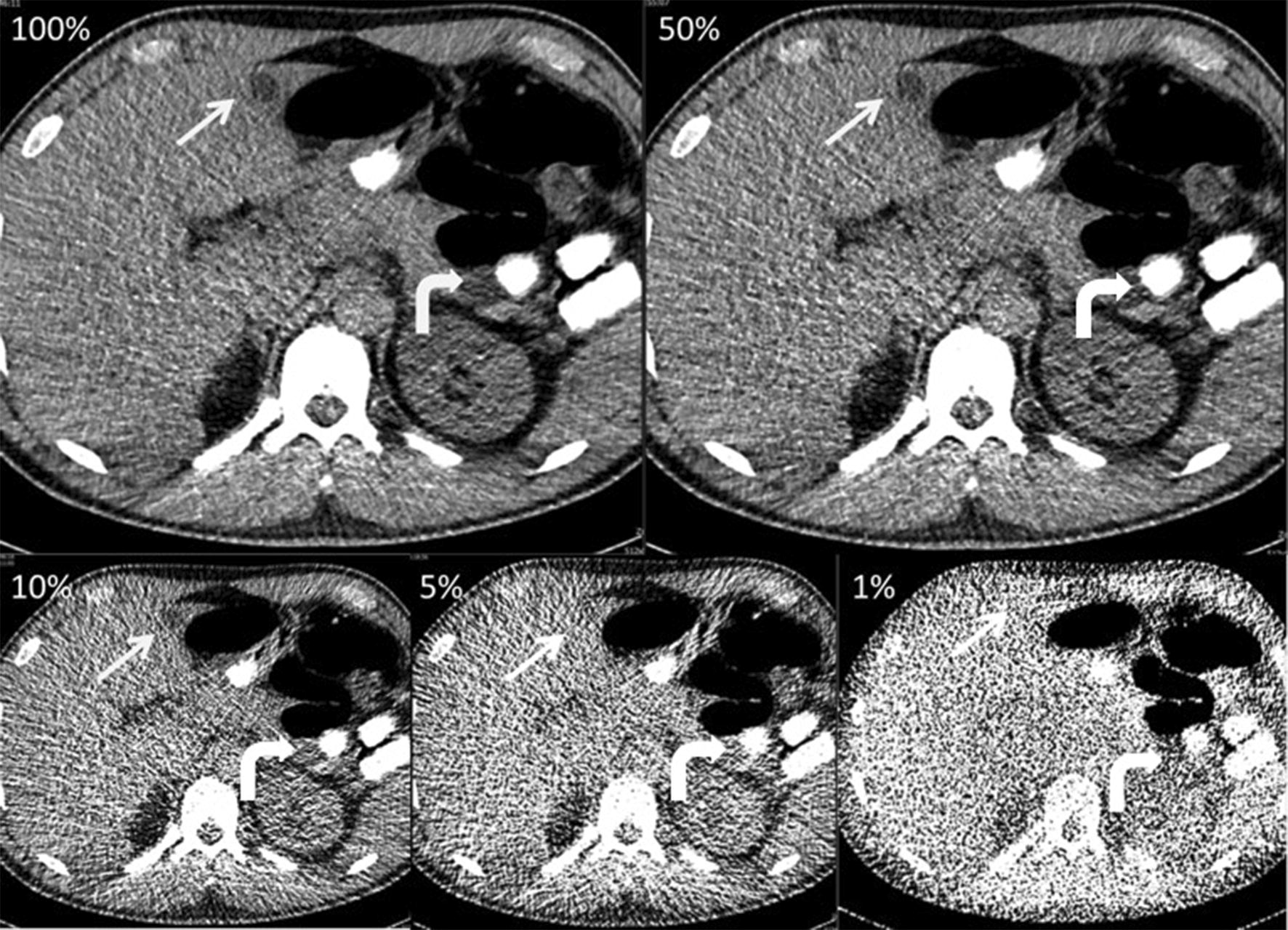
A 40-year-old body packer, who was examined with 80 kVp and automated tube current modulation (tube current time product: 134 mAs, effective dose at 100%: 1.97 mSv). The axial reconstructions (soft tissue window, window level 40/300) with stepwise dose reduction (100%, 50%, 10%, 5%, and 1%) show multiple solid body packets (white curved arrows), which remained easily detectable at only 1% of initial dose (0.02 mSv). The liver cyst (white arrows) was missed by all readers at dose level 5% and 1%

At the radiation-dose levels of 100, 50, and 10%, all incidental findings (12/12, 100%) were detected by both readers (Fig. 3). Detection of incidental findings was significantly worse at 5% (6/12, 50%) and 1% (3/12, 25%) (p < 0.001 for both).

## Discussion

We found abdominal CT for suspected body packer with an effective dose of 1.05 mSV to enable reliable detection of body packets in our collective. Subjective image quality was reduced for all reduced dose levels compared to 100% of the initial radiation dose. In our collective, 10% of the initial dose (0.21 mSV) was required to provide sufficient image quality for the detection of secondary findings.

Individuals in our study were of young age with a mean of 37.8 ± 11.1 years. This is in accordance to previous studies on CT body packing that found individuals suspected of body packing to be mainly young adults [3, 7]. Dose saving in young adults is desirable, however; for reasons of possible body packer complications or legal consequences, the maintenance of a diagnostic image quality is mandatory. Noncontrast CT is superior to plain radiography in the detection of incorporated body packets and currently reflects the reference standard in the detection of ingested drug containers [5, 6]. However, young age and the strict indication due to radiation exposure in otherwise healthy individuals require a focus on radiation-dose optimization. Magnetic resonance imaging (MRI) and Sonography are possible alternatives without radiation exposure. Due to this special setting with armed customer officers and frequently noncompliant delinquents, these methods pose complications during performance in clinical routine. However, MRI and Sonography seem to be suitable alternatives in cases of pregnancy and children [17].

We found an effective dose of 0.11 mSv to be sufficient for the detection of cocaine body packets. Prior studies evaluated body packing CT protocols with reduced radiation dose that are used in clinical routine and reported diagnostic image quality for CT protocols with an effective dose of 1.06–2.05 mSv [5, 8, 11, 18, 19] which is close to an abdominal radiography. However, due to the clinical setting of previous studies, a radiation-dose threshold could not be determined.

To our knowledge, extensive dose reduction in the context of body packing was investigated only by Maurer et al. [9] in an animal model and Laberke et al. in a postmortem study [10]. Maurer et al. [9] investigated a total number of twelve solid cocaine containers, which were introduced into the intestine of crossbred pigs. The pigs underwent repeated CT examinations with a fixed tube voltage of 80 kVp and a stepwise reduction of tube current from 350 to 10 mAs. The threshold for correct detection of all containers was 125 mAs, which has resulted in an effective dose of 1.0 mSv. This is ten times higher than the 0.1 mSv found in our study in humans. Laberke et al. placed up to 20 body packets in the alimentary tract of human cadavers. They showed a threshold of 0.6 mSv for the correct identification of body packets. Due to different postmortem changes in animal and human cadavers, the image interpretation of CT scans can differ compared to living individuals. Therefore, our study is the first study which evaluated an extensive dose reduction in the context of body packing in clinical routine.

We found a mean attenuation of body packets of 292.2 HU. The mean attenuation of body packs in the study by Maurer et al. [9] was − 69.6 HU (range 135–247 HU). The negative values seem to be the reason for the high threshold of 125 mAs for correct identification in the animal study. We did not find any cocaine containers with negative density values and cannot give an explanation for the negative values reported by Maurer et al. [9]. Negative values usually are attributable to the admixture of the content, which is usually not a pure form of cocaine. However, most studies that investigated cocaine body packing on CT reported density values comparable to our results [5, 6, 8, 19–21].

Of note, the noise-insertion tool used herein has been previously validated and allows for a reliable simulation of radiation-dose reduction in CT [12]. This allows for the assessment of radiation-dose thresholds in regards to diagnostic yield in a clinical setting while not exposing patients to additional radiation. Additional CT examinations for research purposes have been reported, and the ethical considerations were discussed [22, 23]. A recent study reported a technique to reconstruct multiple dose levels from a dual-energy CT examination [23]. However, even in this study, an additional CT examination with a not-clinically indicated radiation exposure for the patients was necessary. The noise simulation tool used in our study may help to improve research results for radiation-dose reduction without exposing patients to unnecessary radiation.

We found a significant decline of image quality upon decreasing the radiation dose. At 10% of the initial dose, several CT examinations were rated nondiagnostic based on the European Quality Criteria [13]. However, screening for body packing is a special task, and 10% of the initial dose provided sufficient image quality for detection of all body packets.

It was found in former studies that a Deff of 30 cm correlates with a BMI of 26 in adults undergoing an abdominal CT [15]. As the mean Deff was 29.9 cm in this study, the averaged physical constitution of the investigated individuals can be described as obese. Based on this, the findings of this study regarding relevant dose reduction by preserving diagnostic image quality gain even more significance.

Other techniques like the dual-energy CT have evaluated if heroin and cocaine can be distinguished using dual-energy CT. They showed that the slope of the spectral curve and the DEI from dual-energy CT data can be used to distinguish heroin and cocaine in vitro [24].

All incidental findings were detected with 100%, 50% and 10% of initial dose. At dose reduction level 5% and 1% detection of incidental findings was significantly worse. There is only one study which evaluated incidental findings detected by noncontrast CT scans in the setting of body packing [25]. Overall, they found 31 incidental findings in 18 CT scans. There was no information concerning the performed CT scan protocol. We showed that incidental findings are reliably detectable with an effective dose of 0.21 mSv. The evaluated effective doses are specific to the manufacturer, hardware, and level of iterative reconstruction used in this study as explained in “Patient, materials and methods” section.

Our study has limitations. First, we performed a retrospective study, and the number of individuals with body packets was limited. Moreover, according to the small number of positive cases `reader memory´ effects on the detection of body packs and secondary effects were possible. In addition, a software tool was used to model dose reduction, however, the method has been previously validated. Due to the software limitations, we only reconstructed axial CT images. Second, we only investigated drug containers made from cocaine with a limited range of packet density; however, cocaine is the most frequently concealed drug transported by body packers [3]. Third, our study population did not include very obese individuals for whom our results might not be applicable. Last, stool analysis was only available in cases positive on CT. However, CT is regarded as the reference standard for the detection of body packets and thus we think that the reference standard applied in our study, which was also based on full-dose abdominal CT scans was adequate [5, 6, 9]. All body packing patients had ingested multiple packets. Results may differ for patients with a single packet; however, from our experience, this reflects clinical routine as body packers typically swallow multiple packets.

## Conclusion

Our results indicate that dose of abdominal CT for the detection of intracorporeal cocaine body packets can be markedly reduced to up to 5% of the initial dose while still providing sufficient image quality to detect ingested body packets. However, an effective dose of 0.21 mSv (10% of initial dose) seems to be required to properly identify secondary findings.
